# jmzReader: A Java parser library to process and visualize multiple text and XML-based mass spectrometry data formats

**DOI:** 10.1002/pmic.201100578

**Published:** 2012-04-26

**Authors:** Johannes Griss, Florian Reisinger, Henning Hermjakob, Juan Antonio Vizcaíno

**Affiliations:** 1 EMBL-European Bioinformatics Institute, Wellcome Trust Genome CampusHinxton, Cambridge, UK; 2 Department of Medicine I, Medical University of ViennaVienna, Austria

**Keywords:** Bioinformatics, Data standard, Java, MS data processing, Proteomics standards initiative

## Abstract

We here present the jmzReader library: a collection of Java application programming interfaces (APIs) to parse the most commonly used peak list and XML-based mass spectrometry (MS) data formats: DTA, MS2, MGF, PKL, mzXML, mzData, and mzML (based on the already existing API jmzML). The library is optimized to be used in conjunction with mzIdentML, the recently released standard data format for reporting protein and peptide identifications, developed by the HUPO proteomics standards initiative (PSI). mzIdentML files do not contain spectra data but contain references to different kinds of external MS data files. As a key functionality, all parsers implement a common interface that supports the various methods used by mzIdentML to reference external spectra. Thus, when developing software for mzIdentML, programmers no longer have to support multiple MS data file formats but only this one interface. The library (which includes a viewer) is open source and, together with detailed documentation, can be downloaded from http://code.google.com/p/jmzreader/.

High-throughput mass spectrometry (MS) proteomics experiments can generate huge amounts of data and through new publication guidelines [1] and requirements by funding agencies, more and more data is becoming available in the public domain. This unprecedented availability of MS data comes with great potential that can only be fully harvested if the processing of this data is made as easy as possible. Recently, the Human Proteome Organization (HUPO) proteomics standards initiative (PSI) officially released the second version (v1.1) of mzIdentML [2], the recommended exchange format for peptide and protein identification data. This version will be a stable data standard for several years. We strongly believe that this new standard file format will considerably facilitate the exchange and (re-) processing of MS proteomics data irrespective of the used search engine and analysis software. First software packages supporting mzIdentML already exist, as for example, the Mascot search engine [3] (from version 2.3), converters from various file formats to mzIdentML, such as Sequest result files [4] and Proteome Discoverer (.msf and .protXML files; e.g., within ProCon: http://www.medizinisches-proteom-center.de/ProCon), OMSSA [5], and X!Tandem [6] output files, as well as the recently developed Java application programming interface (API) jmzIdentML (http://jmzidentml.googlecode.com).

However, it is important to highlight that reported identifications in mzIdentML only contain references to the supporting spectra data that can then be found in external MS data files. As mzIdentML files, thereby, do not directly contain spectra information, a major part of a proteomics experiment's data is still only available spread across a multitude of file formats. Among them, mzML is the corresponding PSI standard for MS data [7], which is being adopted at a good pace. Nevertheless, its predecessors, the XML-based formats, mzData and mzXML [8], are still heavily used as well as very popular text-based peak lists formats, such as mascot generic format (MGF), DTA, Micromass PKL, and MS2 [9].

Although a Java library for the mzML format (called jmzML) already exists [10], there are no comparable Java APIs available for the other MS data formats. The jrap library (http://tools.proteomecenter.org/wiki/index.php?title=Software:JRAP), a Java API to access mzXML files developed by the Institute for Systems Biology (ISB, Seattle), is no longer under active development. The same applies to the ProteomeCommons.org IO Framework (http://www.proteomecommons.org/current/531/), which lacks full support of the latest mzXML, mzML, and MS2 formats. In addition, this last framework only provides limited access to the complete information stored in the supported file formats. The majority of other parser libraries focuses on programming languages other than Java such as C/C++/C# or R [11, 12].

Although the adoption of the community standard format, mzML, for representation of MS data is progressing, an efficient and consistent parsing library for vendor-specific file formats, both for legacy data and current data in other formats will still be required for the foreseeable future. We, here, present the jmzReader library: a collection of Java APIs to efficiently process a multitude of MS data formats optimized for the usage with the mzIdentML standard. The jmzReader library currently consists of six independently usable Java APIs: dta-parser, mgf-parser, ms2-parser, mzdata-parser, mzxml-parser, and pkl-parser as well as a wrapper class around the existing jmzML API [10] (see Supporting Information File S1). All of these APIs implement a common interface and were developed based on the file format descriptions found on the Mascot documentation page (http://www.matrixscience.com/help/data_file_help.html) as well as available publications [8, 9] and file format specific project documentations (http://tools.proteomecenter.org/wiki/index.php?title=Formats:mzXML) for mzXML (supporting versions 2.1–3.2) and http://www.psidev.info/index.php?q=node/80#mzdata for mzData (supporting version 1.05). The mzml-wrapper was built around the existing jmzML API implementing the jmzReader interface and thereby adding mzML support to the jmzReader library for convenience purposes. Through the common jmzReader interface, programmers writing support for peak lists referenced in mzIdentML only have to support one interface to access any of the supported seven file formats (Fig. 1). In addition, each of the parsers contains a format specific Java object model that allows access to the whole information contained in the specific file format. Thus, the various parser APIs are not limited to the use with mzIdentML but form a solid basis for any software processing MS data. All APIs are open source, were written in 100% Java and are thus inherently platform independent.

**Figure 1:**
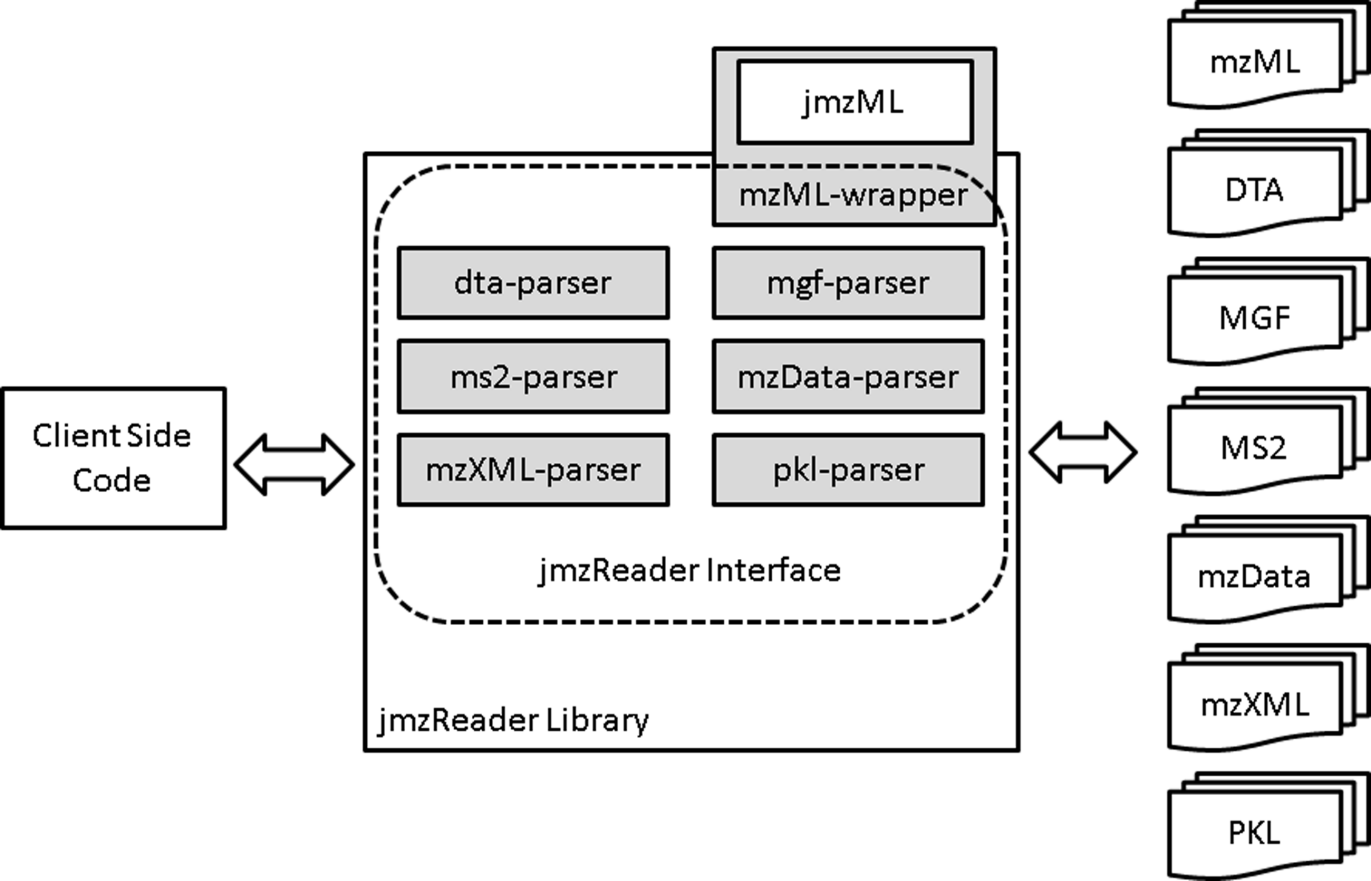
Simplified schema of the jmzReader library's structure displaying its main components and their dependencies. All parsers implement the common jmzReader interface and, in addition, provide a file-format specific Java object model to access a file format's specific information. Thereby, each parser can be used independently of the rest of the jmzReader library. If this detailed information is not required, programmers can process all seven supported file formats using a single, easy-to-use Java interface.

mzXML and mzData are the only XML-based formats among the directly supported file formats. Therefore, the mzXML and mzData parsers were developed using a different technique than the other libraries while still implementing the common jmzReader interface. Similar to the well-established jmzML API, the mzXML and mzData APIs were build using a combination of Java Architecture for XML Binding (JAXB) for generating a Java object model based on an XML schema and the highly efficient xxindex library [10]. xxindex enables the APIs to process arbitrarily large XML files and randomly accessing XML objects within these files without the need to load the whole file into memory. The comfort of a full JAXB Java object model together with the high performance reached through the use of xxindex are seamlessly combined in the mzXML and mzData parser libraries. These technical features are hidden to the users who can completely focus on only using the comfortable Java object model representing the structure of the parsed files without having to worry about any performance issues.

Through the constant enhancement of proteomics techniques the size of the produced data is continuously growing. To tackle this problem each of the APIs within the jmzReader library was optimized for the demands associated with these ever growing file sizes. Every parser provides functions to randomly access spectra within the peak list files without the need to load the whole file into memory. Thereby, arbitrarily large files can be handled independently of the available hardware resources and without a limitation to the API's usability. To circumvent Java's known weak i/o performance a custom wrapper was written around Java's standard class for randomly accessing files *RandomAccessFile* as described by Nick Zhang (http://www.javaworld.com/javaworld/javatips/jw-javatip 26.html). This custom class increased the speed of indexing peak list files by 20 fold and is also available as part of the jmzReader library.

The library's second key feature is its optimized functions to handle mzIdentML's referencing system. Depending on the used file format mzIdentML currently supports three methods to reference spectra in MS data files: through the spectrum id (when available in the format as in mzData, mzXML, and mzML), the spectrum's position in the file (DTA, PKL, MS2, MGF) and, in case of formats where each file can only contain one spectrum (DTA, PKL) through the filename. Detailed information on how mzIdentML references external spectra data can be found in the mzIdentML 1.1 specification document at http://www.psidev.info/index.php?q=node/453. To conveniently access spectra despite these heterogeneous referencing methods every parser implements the function getSpectrumById. This function interprets the passed id based on the underlying file format and structure. Thus, the programmer does not have to implement the various referencing methods available but can use one convenient function only.

The jmzReader library furthermore provides functions to store a MS data file's index in an external source (such as a database). This feature allows the user to efficiently access a given spectrum in a sourcefile without the need to re-index the file again. This function can be used to build a flat-file database able to store significantly larger amounts of MS data than current database systems can efficiently handle. A detailed description of this feature can be found at http://code.google.com/p/jmzreader/wiki/JMzReaderInterface.

All parsers were designed to be used independently of the jmzReader library and provide access to the full information stored in the supported formats. Although text-based formats, such as PKL and DTA, only provide minimalistic information about the underlying MS data mzML as well as mzXML, for example, provide a multitude of metadata. Thus, the common interface had to be designed as a “smallest common denominator.” To still provide users with easy access to the specific features of every file format, each API provides access to the complete information found in the respective file format through a comfortable Java object model. Thereby, the usage of the jmzReader library's APIs is not limited to the use with mzIdentML but provides a solid basis for any software processing MS data in the supported formats. All APIs were developed following a similar design also found in other comparable projects [13–15]. Thereby, the here presented APIs should be straightforward to use even when accessing the more detailed file format specific features. Detailed documentation about the single parser APIs as well as the jmzReader interface together with short code examples can be found at http://code.google.com/p/jmzreader/wiki/Welcome.

The jmzReader library also comes with a simple but powerful, interactive viewer (Fig. 2) to demonstrate the use of the common interface. It can be used to load and view spectra from all supported file formats, simultaneously illustrating the usefulness of the low-memory footprint achieved by the whole library as well as the advantage of the common interface. This viewer can also export any of the loaded MS data files to the commonly used Mascot MGF format.

**Figure 2:**
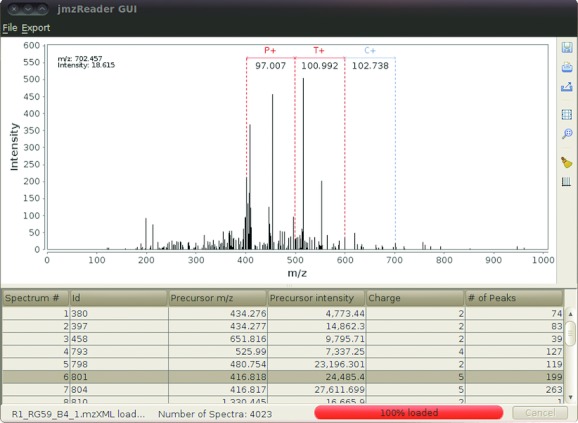
Screenshot of the jmzReader GUI after opening a mzXML file. The selected spectrum is displayed using the mzGraphBrowser library (http://code.google.com/p/pride-toolsuite/wiki/PRIDEmzGraphBrowser). This library allows the user to manually annotate a spectrum and export the loaded file into MGF format. The jmzReader GUI is built using the jmzReader interface and, thus, supports all file formats supported by the jmzReader library.

The PSI proteomics informatics (PI) workgroup is currently developing a complementary standard format for reporting quantification data, called mzQuantML (http://code.google.com/p/mzquantml/). mzQuantML will use the same method for referencing external spectra as the one used in mzIdentML. The jmzReader library will thus also be an ideal foundation for software developments based on mzQuantML. The jmzReader library as well as the independently usable file format specific APIs are already successfully used as basis for the development of several tools and will be a cornerstone of the next version of the PRIDE database [16]. The whole jmzReader library is freely available, and is released as open source under the permissive Apache 2.0 license. The binaries, source code and documentation can be downloaded from the project web site at http://code.google.com/p/jmzreader/.

## Abbreviations

API: application programming interface
GUI: graphical user interface
JAXB: Java architecture for XML binding
PRIDE: PRoteomics IDEntifications (database)
PSI: (Human Proteome Organization) proteomics standards initiative

## Supplementary material

Disclaimer: Supplementary materials have been peer-reviewed but not copyedited.
