# Supplementary material for: jmzReader: A Java parser library to process and visualize multiple text and XML-based mass spectrometry data formats
Source: Proteomics. 2012 Apr 26;12(6):795–8. doi: 10.1002/pmic.201100578 (PMC3472022; doi:10.1002/pmic.201100578)
Supplement: Supplementary file 1 [file pmic0012-0795-SD1.doc]

**jmzReader Library Technical Implementation**

**System Requirements:**

- Java: JRE 1.5+
- Platform: Tested on Mac OS X, Linux, and Windows (XP, Server 2003, 7)

**Website & Source Code:**

<http://code.google.com/p/jmzreader>

**Included Open Source Libraries**

The following libraries are part of the jmzReader library but can also be used separately.

| **dta-parser** | |
| --- | --- |
| **Website & Source Code** | <http://code.google.com/p/jmzreader/wiki/DtaParser> |
| **Description** | The dta-parser library can be used parse DTA files. Peak-list files are indexed and thus do not have to be loaded into memory completely. The library supports concatenated DTA files as well as multiple DTA files in one directory only containing a single spectrum each. |
| **License** | Apache 2 open source license |
| **Language** | Java |
| **External libraries** | BufferedLineReader [http://code.google.com/p/peak-list-parser/wiki/BufferedLineReader]: Parsing files line by line |

| **mgf-parser** | |
| --- | --- |
| **Website & Source Code** | <http://code.google.com/p/jmzreader/wiki/MgfParser> |
| **Description** | The mgf-parser library can be used parse MGF files. MGF files are indexed and thus do not have to be loaded completely into memory. The mgf-parser includes a Java object model providing access to the complete data available in an MGF file. The mgf-parser library furthermore supports writing MGF files. |
| **License** | Apache 2 open source license |
| **Language** | Java |
| **External libraries** | BufferedLineReader [http://code.google.com/p/peak-list-parser/wiki/BufferedLineReader]: Parsing files line by line |

| **ms2-parser** | |
| --- | --- |
| **Website & Source Code** | <http://code.google.com/p/jmzreader/wiki/Ms2Parser> |
| **Description** | The ms2-parser library can be used parse MS2 files. MS2 files are indexed and thus do not have to be loaded into memory completely. The ms2-parser includes a Java object model providing access to the complete data available in an MS2 file. |
| **License** | Apache 2 open source license |
| **Language** | Java |
| **External libraries** | BufferedLineReader [http://code.google.com/p/peak-list-parser/wiki/BufferedLineReader]: Parsing files line by line |

| **mzdata-parser** | |
| --- | --- |
| **Website & Source Code** | <http://code.google.com/p/jmzreader/wiki/MzDataParser> |
| **Description** | The mzdata-parser can be used to parse mzData files. mzData is the PSI’s previous standard file format for MS spectra data. The mzdata-parser library indexes mzData files first using xxindex and thus can provide random access to all elements of an mzData file without having to load the whole file into memory. |
| **License** | Apache 2 open source license |
| **Language** | Java |
| **External libraries** | XXIndex [<http://code.google.com/p/pride-toolsuite/wiki/XXIndex>]: Indexing XML files. |
|  | JAXB [<http://jaxb.java.net/>]: Parsing XML snippets into object model. |

| **mzdata-parser** | |
| --- | --- |
| **Website & Source Code** | <http://code.google.com/p/jmzreader/wiki/MzXmlParser> |
| **Description** | The mzxml-parser can be used to parse mzXML files. mzXML files are the spectra data format used by the Trans Proteomics Pipeline. The mzxml-parser can handle mzXML files starting from version 2.0 up until version 3.2. The mzxml-parser uses the xxindex library to index mzXML files. Thereby, the mzxml-parser can provide random access to all elements of an mzXML file without having to load the whole file into memory. |
| **License** | Apache 2 open source license |
| **Language** | Java |
| **External libraries** | XXIndex [<http://code.google.com/p/pride-toolsuite/wiki/XXIndex>]: Indexing XML files. |
|  | JAXB [<http://jaxb.java.net/>]: Parsing XML snippets into object model. |

| **pkl-parser** | |
| --- | --- |
| **Website & Source Code** | <http://code.google.com/p/jmzreader/wiki/PklParser> |
| **Description** | The pkl-parser library can be used parse PKL files. Peak-list files are indexed and thus do not have to be loaded into memory completely. The library supports concatenated PKL files as well as multiple PKL files in one directory only containing a single spectrum each. |
| **License** | Apache 2 open source license |
| **Language** | Java |
| **External libraries** | BufferedLineReader [http://code.google.com/p/peak-list-parser/wiki/BufferedLineReader]: Parsing files line by line |

| **BufferedRandomAccessFile** | |
| --- | --- |
| **Website & Source Code** | http://code.google.com/p/jmzreader/wiki/BufferedRandomAccessFile |
| **Description** | Java has a known low i/o performance that is especially striking in its standard class for randomly accessing files RandomAccessFile. To provide random access to the text based file formats (such as DTA, MS2, etc.) all files need to be pre-scanned line by line and the position of the spectra in the file saved in an index. To circumvent the very low performance of RandomAccessFile's readLine function all parsers use the BufferedRandomAccessFile class as described by Nick Zhang (http://www.javaworld.com/javaworld/javatips/jw-javatip26.html). |
| **License** | Apache 2 open source license |
| **Language** | Java |
