# Supplementary material for: jmzReader: A Java parser library to process and visualize multiple text and XML-based mass spectrometry data formats
Source: Proteomics. 2012 Apr 26;12(6):795–8. doi: 10.1002/pmic.201100578 (PMC3472022; doi:10.1002/pmic.201100578)
Supplement: Supplementary file 2 [file pmic0012-0795-SD2.pdf]

Source Code Example

The following example shows how the jmzReader library can be used in conjunction with the jmzIdentML API (http://code.google.com/p/jmzidentml/).

This example is based on the *55merge_omssa.mzid* file and its peak list file *55merge.mgf*. Both files can be found in the jmzReader download section (http://code.google.com/p/jmzreader/downloads/list).

```
/**
 * This example ignores any exception and error handling.
 * Furthermore, this example assumes that the corresponding
 * peak list file for the given mzIdentML file is already
 * known.
 */

JMzReader jmzreader = new MgfFile(new File("/path/to/55merge.mgf"));
MzIdentMLUnmarshaller unmarshaller = new MzIdentMLUnmarshaller(new
URL("/path/to/55merge_omssa.mzid"));

List<SpectrumIdentificationList> sil = ad.getSpectrumIdentificationList();

for (SpectrumIdentificationList sIdentList : sil) {
    for (SpectrumIdentificationResult spectrumIdentResult
            : sIdentList.getSpectrumIdentificationResult()) {

        // get the spectrum's id in the referenced file
        String spectrumID =  spectrumIdentResult.getSpectrumID(); // this
returns a value like "index=246"
        // this is the actual reference to the spectrum file
        // but as in this example the spectrum file is already
        // known it is not needed. In real world examples this
        // reference would be used to identify the source spectrum
        // file
        String spectrumFileRef = spectrumIdentResult.getSpectraDataRef();

        // as MGF files are index based the "index=" portion of the spectrumId
needs to be removed
        String spectrumIndex = spectrumID.substring(6);

        // using the index the spectrum can now be retrieved from the
        // MGF file.
        Spectrum spectrum = jmzreader.getSpectrumById(spectrumIndex);
    } // end spectrum identification results
}
```
